# Biomechanical Study of Arthroscopic All-Inside Anterior Talofibular Ligament Suture Augmentation Repair, Plus Suture Augmentation Repair and Anterior Tibiofibular Ligament’s Distal Fascicle Transfer Augmentation Repair

**DOI:** 10.3390/jcm11175235

**Published:** 2022-09-05

**Authors:** Lei Xiao, Boyuan Zheng, Yijuan Zhou, Dahai Hu, Jieruo Li, Xiaofei Zheng, Huige Hou, Huajun Wang

**Affiliations:** 1 Department of Bone and Joint Surgery and Sports Medicine Center, The First Affiliated Hospital, Jinan University, Guangzhou 510630, China; 2 Department of Emergency, The First Affiliated Hospital, Jinan University, Guangzhou 510632, China; 3 International School, Jinan University, Guangzhou 510632, China

**Keywords:** ankle arthroscopy, anterior talofibular ligament, suture augmentation repair, plus suture augmentation repair, ATiFL-DF transfer augmentation repair, biomechanics

## Abstract

**Objective:** To explore the biomechanical efficacy of arthroscopic all-inside anterior talofibular ligament (ATFL) suture augmentation repair, plus suture augmentation repair and anterior tibiofibular ligament-distal fascicle (ATiFL-DF) transfer augmentation repair, so as to provide a basis for the accurate selection of ATFL repair in clinical practice. **Methods:** Twenty-four (12 pairs) fresh frozen human cadaver ankle specimens were used. Six of the ankle specimens were set as the normal group, and the other 18 ankle specimens were used to establish ATFL injury models. The ATFL was then repaired using arthroscopic all-inside ATFL suture augmentation repair (suture augmentation group), plus suture augmentation repair (plus suture augmentation group) and ATiFL-DF transfer augmentation repair (biological augmentation group), respectively. After the repaired ATFL was separated, the ankle specimens were fixed on an electronic universal testing machine with a customized fixture for the tensile test, and the ultimate failure load (N) and stiffness (N/mm) of the ankle specimens were compared. **Results:** The ultimate failure load of the plus suture augmentation group (229.3 ± 66.7 N) was significantly higher than that in the normal group (148.2 ± 39.4 N, *p* = 0.045) and the biological augmentation group (131.3 ± 38.8 N, *p* = 0.013). There was no statistical difference in ultimate failure load between the suture augmentation group (167.2 ± 47.2 N), the normal group and the biological augmentation group. The stiffness of the plus suture augmentation group (26.2 ± 8.2 N/mm) was significantly higher than that in the normal group (12.1 ± 3.8 N/mm, *p* = 0.005) and the biological augmentation group (12.7 ± 5.2 N/mm, *p* = 0.007). The stiffness of the suture augmentation group (23.6 ± 7.0 N/mm) was significantly higher than that in the normal group (*p* = 0.024) and the biological augmentation group (*p* = 0.033). There was no statistical difference in stiffness between the plus suture augmentation group and the suture augmentation group, and no statistical difference in stiffness between the normal group and the biological augmentation group. **Conclusions:** The tensile strength and rigidity of plus suture augmentation repair were significantly better than those of normal ATFL, suture augmentation repair and ATiFL-DF transfer augmentation repair. Suture augmentation repair can obtain tensile strength similar to normal ATFL and ATiFL-DF transfer augmentation repair, and suture augmentation repair can obtain rigidity significantly better than normal ATFL and ATiFL-DF transfer augmentation repair. ATiFL-DF transfer augmentation repair can obtain tensile strength and rigidity similar to normal ATFL.

## 1. Introduction

Ankle sprain is one of the most common sports injuries, especially in basketball and football, and the incidence can be as high as 45% and 31% of all sports injuries, respectively [1,2]. Once chronic lateral ankle instability (CLAI) develops, surgical treatment is often required. In the last ten years, with the development of surgical instruments and technology, the technology of ankle arthroscopic repair of the anterior talofibular ligament (ATFL) has developed rapidly [3,4]. The application of wire anchors [5], knotless anchors [6], lasso ring techniques and the safety zone concept [7,8] in ankle arthroscopy solves many of the previous problems to some extent. At the same time, with the emergence of various new technologies, the repair of ATFL under total ankle arthroscopy is still developing [9,10,11,12,13,14]. Vega et al. [15] performed ATFL suture augmentation repair under total ankle arthroscopy in 15 patients with CLAI with poor residual ligament tissue quality in 2018, and all patients achieved definite curative effects. In this total arthroscopic repair, the stump of the ATFL is sutured to the attachment and then augmented with a high-strength suture connecting the fibula to the side of the talus. This is the first report of ATFL suture augmentation repair under arthroscopy anywhere in the world. At present, there is still a lack of relevant biomechanical research. After learning the operation, the Department of Sports Medicine of our hospital has widely used it in clinic. At the same time, for some patients with high exercise demand, we made improvements on the basis of suture augmentation repair. On the basis of suture augmentation repair, we perform another suture enhancement; that is, use two (four strands) sutures to enhance and repair the ATFL. We name this operation “plus suture augmentation repair”. As far as we know, this operation has not been reported at home or abroad. In 2019, Vega et al. [16] completed ATFL anterior tibiofibular ligament-distal fascicle (ATiFL-DF) transfer augmentation repair under total arthroscopy on the ankles of five cadavers. Compared with the above two repairs, ATiFL-DF was used instead of suture for augmented repair in this anatomical study. This anatomical study confirmed that it is safe and feasible to practice ATFL ATiFL-DF transfer augmentation repair under total arthroscopy, but there is still a lack of relevant biomechanical research.

Therefore, in our study, after establishing most ATFL injury models (residual ligaments: 25–50%) on ankle specimens, suture augmentation repair, plus suture augmentation repair and ATiFL-DF transfer augmentation repair under total arthroscopy were, respectively, used to repair the ATFL, and the biomechanical properties of the ATFL after each surgical repair were compared by a pull-out test experiment. It provides a basis for the accurate selection of ATFL repair for injury patients in clinical operation.

## 2. Materials and Methods

### 2.1. Specimen

Twenty-four (12 pairs) fresh frozen human cadaver ankle specimens were used. The average age of the specimens was 55.7 years (39–67 years). A difference of >30% is assumed to be significant. According to the a priori power analysis, 6 ankle specimens are needed in each group, with a difference of 30%, a standard error of 15%, a power of 0.8 and a significance level of 0.05. Inclusion criteria: (1) there was no ankle ligament tear in the ankle of the cadavers, (2) did not undergo ankle surgery, (3) died of non-cancer diseases. Exclusion criteria: (1) Age < 20 years old or >70 years old, (2) had ankle injury. Ankle specimens were stored at −20° and thawed at room temperature 24 h before use. Six ankle specimens were randomly divided into normal group. In 18 ankle specimens, most ATFL injury models were established (residual ligament: 25–50%), and ATFL suture augmentation repair (suture augmentation group), plus suture augmentation repair (plus suture augmentation group) and ATiFL-DF transfer augmentation repair (biological augmentation group) were performed under total arthroscopy, respectively. The study was approved by the Ethics Committee of the First Affiliated Hospital of Jinan University.

### 2.2. Establishment of Most ATFL Injury Models (Residual Ligament: 25–50%)

**Figure 1:**
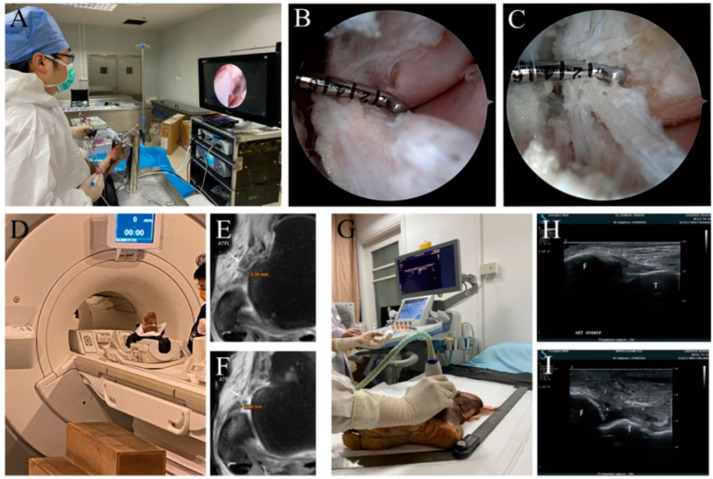
Establishment of most ATFL injury models. (**A**–**C**): the severe ATFL injury model was established under total arthroscopy. (**D**–**F**): MRI confirmed successful modeling before and after operation. (**G**–**I**): B-ultrasound confirmed successful modeling before and after operation. MRI: magnetic resonance imaging.

Before establishing the model, the ankle specimens were detected by magnetic resonance and B-ultrasound, respectively. While further verifying that the selected ankle specimens were consistent with the included specimens, the width of the ATFL in the ankle specimens was recorded. In order to simulate severe ATFL injury, specimens underwent partial ATFL transection under ankle arthroscopy to render the residual ligament 25–50% of the original. The operation was as follows: Instruments included: 4.0 mm Kirschner wire, 4.0 mm 30° arthroscopy, 3.5 mm arthroscopic electric planer, 3.5 mm electrocoagulation/electrocautery arthroscopic electric knife and standard ankle arthroscopic instruments. The ankle specimen was fixed on a custom bench support by using a 4.0 mm Kirschner wire. A marking pen was used to mark the surface projection positions of the safety area, ATFL and fibula on the body surface of the ankle joint. The ankle dorsiflexion technique without stretching was used, and three approaches were established: the anteromedial approach, anterolateral approach and anterolateral auxiliary approach. The anteromedial approach was established at the level of the ankle line and close to the medial side of the anterior tibial muscle tendon, the anterolateral approach was established at the level of 0.5–1.0 cm near the proximal end of the ankle line and close to the lateral side of the third peroneal muscle tendon, and the anterolateral auxiliary approach was established at the level of 0.5–1.0 cm at the distal end of the ankle line and close to the lateral side of the third peroneal muscle tendon. Arthroscopic observation of the internal structure of the ankle joint reconfirmed that the specimens met the inclusion criteria and exclusion criteria. The planer was used to properly remove the synovial tissue, the upper bundle branch of the ATFL and its fibular attachment point were identified, the electrocoagulation/electrocautery arthroscopic knife was introduced through the anterolateral proximal approach, and the electrocoagulation/electrocautery arthroscopic knife was used to separate the upper bundle branch of the ATFL from the fibular attachment point from the proximal end to the distal end. During separation, the end scale of the probe hook was continuously used for comparison to ensure that residual ligament was 25–50% of the original. After the operation, magnetic resonance and B-ultrasound were performed again. The successful modeling was confirmed by comparing the width of the ATFL of ankle specimens before and after the operation. This operation was performed by the same senior orthopedic doctor (see Figure 1).

### 2.3. Operative Method

#### 2.3.1. ATFL Suture Augmentation Repair under Total Arthroscopy

**Figure 2:**
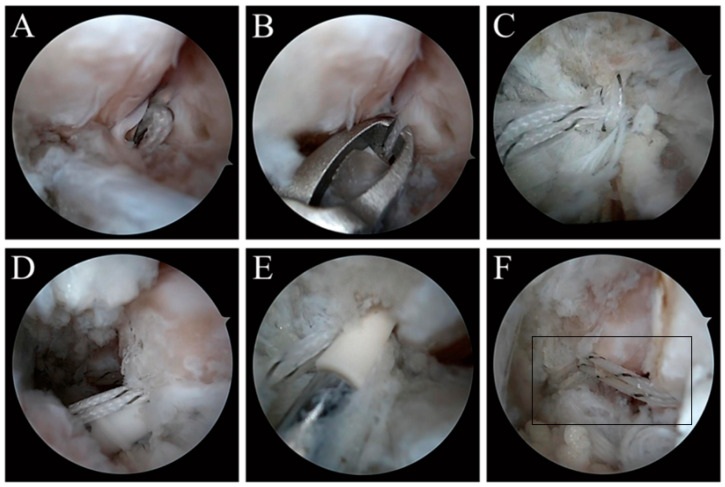
ATFL suture augmentation repair under total arthroscopy. (**A**): Line crossing. (**B**): Line grasping. (**C**): Ring sleeve. (**D**): The fibula side was implanted with knotless anchor. (**E**): The talus side was implanted with knotless anchor. (**F**): Postoperative effect.

Instruments included: 4.0 mm 30° arthroscopy, 3.5 mm arthroscopic electric planer, 3.5 mm electrocoagulation/electrosurgical arthroscopic electric knife, lumbar puncture needle, high resistance and non-absorbable No. 0 line (Fiberwire, Arthrex, Naples, FL, USA), two knotless anchors (Pushlock 2.9 mm × 15 mm, Arthrex, Naples, FL, USA) and standard ankle arthroscopic instruments. A marking pen was used to mark the surface projection positions of the safety area, ATFL and fibula on the body surface of the ankle joint, and three approaches were established: the anteromedial approach, anterolateral approach and anterolateral auxiliary approach. The anteromedial approach was established at the level of the ankle line and close to the medial side of the anterior tibial muscle tendon, the anterolateral approach was established at the level of 0.5 cm at the distal end of the ankle line and close to the lateral side of the third peroneal muscle tendon, and the anterolateral auxiliary approach was established at the level of 0.5 cm at the distal end of the ankle line, close to the anterior side of the fibula and 1.0 cm away from the fibular tip. Under arthroscopy, ATFL residue was 25–50% of the original. When the ankle is in dorsiflexion, many structures near the lateral region of the ankle are observed and identified: the anterior side of the distal portion of the fibula, the lateral wall of the talus, the joint capsule, the upper fascicle of the ATFL and the talus neck. Through the anterolateral approach, the synovium was cleaned with an electric planer to show the fibular attachment footprint of the ATFL. The No. 0 suture was folded in half after passing through the lumbar puncture needle, the lumbar puncture needle was introduced through the anterolateral approach, and then the lumbar puncture needle was passed through the ATFL from the lateral to the medial under the direct vision of arthroscopy. Then, with the help of the arthroscopic gripper, the No. 0 suture was captured through the anterolateral auxiliary approach, where the folded suture ends formed a ferrule. A thread grabber was used to pull both ends of the suture through the ferrule and pull out from the anterolateral auxiliary approach. Both ends of the suture were tightened. It could be seen that the ligament was firmly grasped by the suture. The fibular footprints or slightly distal ends of the ATFL should be selected as far as possible. The direction of the drill bit was from front to back, parallel to the plantar plane and the lateral sulcus plane. After drilling, the suture was threaded through the anchor. Before inserting the anchor, the tension of the suture was fully adjusted, and the anchor was introduced into the hole by knocking in the dorsiflexion and valgus position of the ankle. After the anchor was implanted, both ends of the suture were not cut. The thread grabber was used to pull out the suture from the anterolateral approach. The talus lateral anchor was identified near the talus neck by arthroscopy. The drill guide was inserted through the anterolateral approach and placed in the center of the talus neck; that is, in front of the talus attachment point of the ATFL. The drill bit should be oriented from the distal end of the talus to the proximal end to avoid invading the subtalar joint space and point to the medial malleolus. After drilling, the suture was threaded through the anchor and the anchor placed by knocking. Since the purpose of suture augmentation repair is only to protect the repaired ligament, it should be handled with caution and should not be too tight. To avoid this, the ankle should be in a neutral position when the anchor is implanted, rather than valgus or dorsiflexion. Finally, the suture stump was clipped at the lateral anchors of the talus and the incision was closed (Figure 2).

#### 2.3.2. ATFL Plus Suture Augmentation Repair under Total Arthroscopy

**Figure 3:**
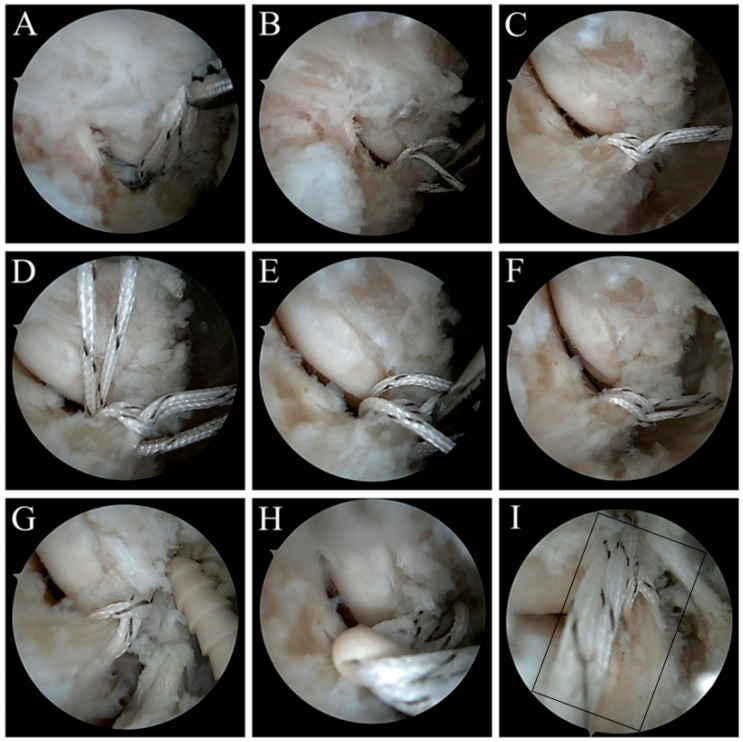
ATFL plus suture augmentation repair under total arthroscopy. (**A**): Line crossing and line grasping. (**B**,**C**): Ring sleeve. (**D**–**F**): Line crossing, line grasping and ring sleeve around the ATFL again. (**G**): The fibula side was implanted with knotless anchor. (**H**): The talus side was implanted with knotless anchor. (**I**): Postoperative effect.

The operation method of ATFL plus suture augmentation repair under total arthroscopy is basically the same as that of ATFL suture augmentation repair under total arthroscopy. The main differences are as follows: 1. The plus suture augmentation repair uses two highly resistant and non-absorbable No. 0 lines. First use one line to pass through the loop around the ATFL, and then use another line to repeat the loop around the ATFL. 2. When placing the anchor at the fibula side anchor and talus side anchor, pass 2 wires (4 strands) through the anchor (Figure 3).

#### 2.3.3. ATFL ATiFL-DF Transfer Augmentation Repair under Total Arthroscopy

**Figure 4:**
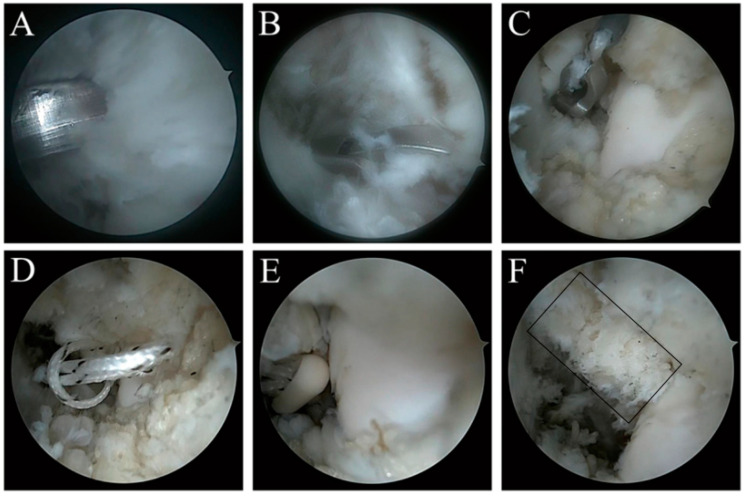
ATFL ATiFL-DF transfer augmentation repair under total arthroscopy. (**A**,**B**): ATiFL-DF was separated from the lateral tibial insertion by osteotomy. (**C**): The stapler passes through the free ATiFL-DF. (**D**): The ring is sleeved with the free ATiFL-DF. (**E**): The talus side was implanted with knotless anchor. (**F**): Postoperative effect.

Instruments included: 4.0 mm 30° arthroscopy, 3.5 mm arthroscopic electric planer, 3.5 mm electrocoagulation/electrosurgical arthroscopic electric knife, lumbar puncture needle, high resistance and non-absorbable No. 0 line (Fiberwire, Arthrex, Naples, FL, USA), a knotless anchor (Pushlock 2.9 mm × 15 mm, Arthrex, Naples, FL, USA) and standard ankle arthroscopic instruments. A marking pen was used to mark the surface projection positions of the safety area, ATFL and fibula on the body surface of the ankle joint, and to guard against superficial peroneal nerve injury. The ankle dorsiflexion technique without stretching was used, and three approaches were established: the anteromedial approach, anterolateral approach and anterolateral auxiliary approach. The anteromedial approach was established at the level of the ankle line and close to the medial side of the anterior tibial muscle tendon, the anterolateral approach was established at the level of 0.5 cm at the distal end of the ankle line and close to the lateral side of the third peroneal muscle tendon, and the anterolateral auxiliary approach was established at the level of 0.5 cm at the distal end of the ankle line, close to the anterior side of the fibula and 1.0 cm away from the fibular tip. Under arthroscopy, ATFL residue was 25–50% of the original. When the ankle is in dorsiflexion, many structures near the lateral region of the ankle are observed and identified: the anterior side of the distal portion of the fibula, the lateral wall of the talus, the joint capsule, the upper fascicle of the ATFL and the talus neck. The osteotomy device was introduced through the anterolateral approach and the ATiFL-DF was separated from the tibial insertion with small bone fragments from the insertion. Once the ligament was separated from the tibia, the ATiFL-DF was bent downward with the help of grasping the suture and probe. The No. 0 suture was folded in half after passing through the lumbar puncture needle, the lumbar puncture needle was introduced through the anterolateral approach, and then the lumbar puncture needle was passed through the ATiFL-DF from the lateral to the medial under the direct vision of arthroscopy. Then, with the help of the arthroscopic gripper, the No. 0 suture was captured through the anterolateral auxiliary approach, where the folded suture ends formed a ferrule. Using a thread grabber, both ends of the suture were pulled through the ferrule and pulled out from the anterolateral auxiliary approach. Both ends of the suture were tightened. It could be seen that the ATiFL-DF was firmly grasped by the suture. The anchor point on the talus should be selected at the talus neck, and the anatomical mark is the exposed triangular area located at the anterolateral edge of the talus. The talus neck was cleaned with a planer to prepare for drilling. The drill guide was introduced through the anterolateral distal approach, and centered on this position, the drill bit pointed to the tip of the medial malleolus. After drilling, the suture was threaded through the anchor. After adjusting the tension of the suture, the anchor was placed by knocking. Finally, the suture stump was clipped at the lateral anchors of the talus and the incision was closed (Figure 4).

### 2.4. Separation of ATFL

**Figure 5:**
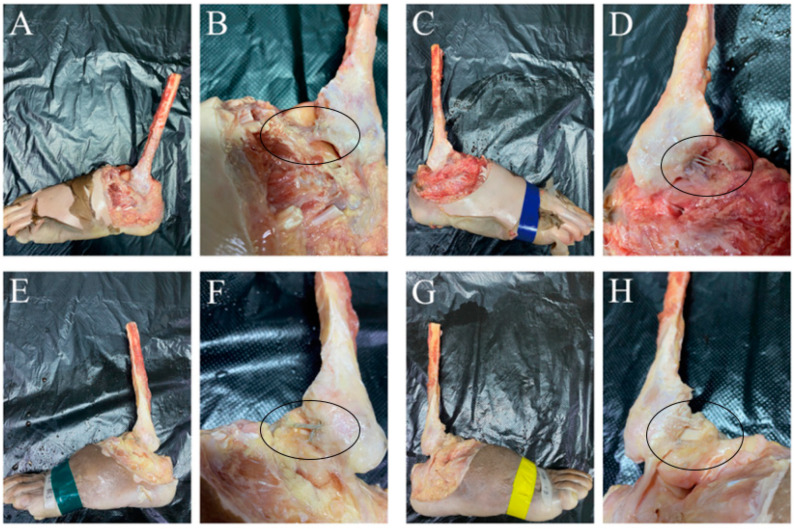
Separation of ATFL. (**A**,**B**): Ankle specimens of the normal group after separation. (**C**,**D**): Ankle specimens of the suture augmentation group after separation. (**E**,**F**): Ankle specimens of the plus suture augmentation group after separation. (**G**,**H**): Ankle specimens of the biological augmentation group after separation.

After the repair of the ATFL, the ATFL of all 24 ankle specimens were separated by a unified method, and all were completed by the same surgeon. In addition to the ATFL attachment from the distal fibula to the lateral talus neck, the soft tissue and muscle on the tibia and fibula were completely removed. The feet and skin were kept intact. An oval incision was made in the middle of the foot and extended backward to the Achilles tendon stop. After resection of soft tissue, deltoid ligament, anterior capsule and posterior capsule were cut, leaving only intact lateral ligament. The combined ligament, posterior talofibular ligament and CFL were removed, and the tibia was removed. Only the fibula and ATFL were separated (Figure 5).

### 2.5. Biomechanical Test

**Figure 6:**
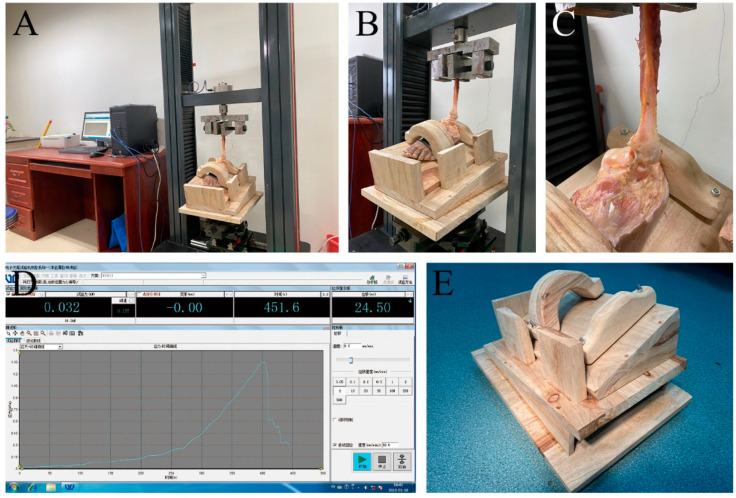
Pull-out experiment. (**A**–**C**): Schematic diagram of pull-out experiment. (**D**): Schematic diagram of software work. (**E**): Custom platform.

A custom-made platform immobilized the foot at 20° varus and 10° plantar flexion mimicked the physiological condition of ATFL tension and provided a biomechanical test for the worst case. The foot was firmly fixed with wooden foot straps across the instep, and rigid supports on the underside, inside and back were used to restrain the foot and prevent its movement. The ATFL was kept in a uniform line with the load actuator of the electronic universal testing machine and the ankle joint of the specimen and the customized platform were installed on the base of the electronic universal testing machine. The electronic universal testing machine is used for biomechanical testing. The testing machine was calibrated to a load accuracy of ±0.25%. The tensile load was gradually applied with a preload of 15 N for 10 s. The load was then maintained at 15 N for 5 s to eliminate potential creep. The actuator then stretched the ATFL at a speed of 200 mm/min to dissociation. Failure tension was defined as the maximum load sustained during the test. The stiffness was calculated as the slope of the linear region of the load extension curve tangent to the steepest line of the curve. Both the tendon and the repair site were kept wet throughout the experiment. Studies have shown that the biomechanical properties of tissues may change negatively with the drying of specimens. The instrument is an electronic universal testing machine (model ATES6010, Guangzhou Aojin Industrail Automation Systems Co. Ltd., Guangzhou, China), and the software (Guangzhou Aojin Industrail Automation Systems Co. Ltd., Guangzhou, China) is an electronic universal testing machine measurement and control system (V8.3.2) (Figure 6).

### 2.6. Statistical Analysis

SPSS 22.0 was used for statistical analysis, and the Shapiro–Wilk test was used to verify that the data accorded with the normal distribution. The results were expressed as mean ± standard deviation (±SD), and the differences between groups were analyzed by one-way ANOVA. For the analysis of variance with statistically significant difference, the post-Tukey significant difference test was carried out, and *p* < 0.05 was considered to be statistically significant.

## 3. Results

**Figure 7:**
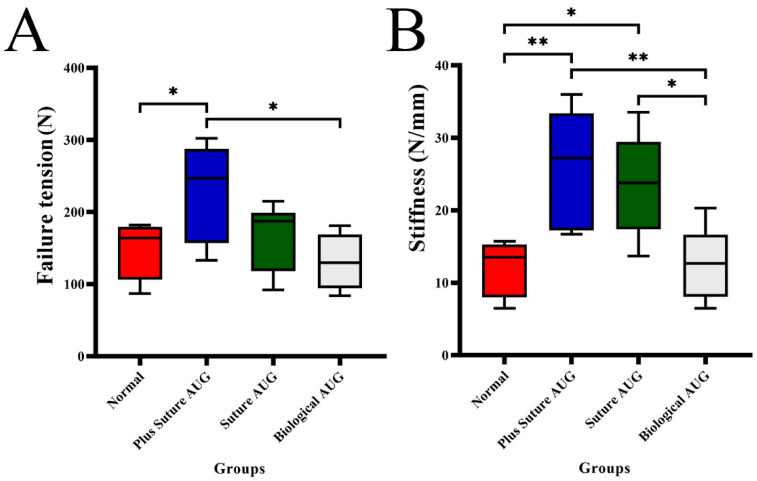
Comparison box diagram of failure tension and stiffness for four groups. The horizontal line represents the median, the box extends from the 25th percentile to the 75th percentile, and the bar represents the maximum and minimum of the observed value. (**A**) The box diagram of failure tension for four groups of ankle specimens. (**B**) The box diagram of stiffness for four groups of ankle specimens. * *p* ˂ 0.05; ** *p* ˂ 0.01.

The failure tension (229.3 ± 66.7 N) of ankle specimens in the plus suture augmentation group was significantly higher than that of the normal group (148.2 ± 39.4 N, *p* = 0.045) and biological augmentation group (131.3 ± 38.8 N, *p* = 0.013). There was no significant difference in the failure tension between the suture augmentation group (167.2 ± 47.2 N), normal group and biological augmentation group, and there was no significant difference in the effective tension between the plus suture augmentation group and suture augmentation group. The stiffness of ankle specimens in the plus suture augmentation group (26.2 ± 8.2 N/mm) was significantly higher than that of the normal group (12.1 ± 3.8 N/mm, *p* = 0.005) and biological augmentation group (12.7 ± 5.2 N/mm, *p* = 0.007). The stiffness of ankle specimens in suture augmentation group (23.6 ± 7.0 N/mm) was significantly higher than that of the normal group (*p* = 0.024) and biological augmentation group (*p* = 0.033). There was no significant difference in the stiffness of ankle specimens between the plus suture augmentation group and the suture augmentation group, and there was no significant difference in the stiffness of ankle specimens between the normal group and the biological augmentation group (Table 1, Figure 7). This section may be divided by subheadings. It should provide a concise and precise description of the experimental results, their interpretation, as well as the experimental conclusions that can be drawn.

## 4. Discussion

In the last ten years, with the development of surgical instruments and techniques, ankle arthroscopic ATFL repair has developed rapidly. The application of wire anchors [5], knotless anchors [6], lasso ring techniques and the safety zone concept [7,8] in ankle arthroscopy solves many of the previous problems to some extent. At the same time, with the emergence of various new technologies, the repair of the ATFL under total arthroscopy is still developing. In recent years, many new techniques of total arthroscopic ATFL repair have been proposed, but there is a lack of relevant biomechanical research. Vega et al. [15] performed total arthroscopic ATFL suture augmentation repair in 15 patients with poor residual ligament tissue quality in 2018. In this total arthroscopic repair, the stump of the ATFL is sutured to the attachment and then augmented with the high-strength suture connecting the fibula to the side of the talus. All patients reported subjective improvement after operation, and the median AOFAS score increased from 66 (44–87) before operation to 100 (85–100) at final follow-up. The results confirmed that ATFL suture augmentation repair under total arthroscopy can achieve very satisfactory results in patients with poor quality of residual ligament tissue. This is the first report of arthroscopic ATFL suture augmentation repair in the world. The ankle arthroscopy team of the Sports Department in our hospital has made some improvements while learning the new technology. Compared with the one suture (two strands) suture augmentation repair reported by Vega et al., we used two sutures (four strands) for suture augmentation and named it “plus suture augmentation repair”. This improved operation has not yet been reported at home or abroad. We believe that plus suture augmentation repair has stronger biomechanical strength than suture augmentation repair, but there is a lack of relevant biomechanical research. In 2019, Vega et al. [16] completed ATFL ATiFL-DF transfer augmentation repair under total arthroscopy on the ankles of five cadavers. Compared with the above two repairs, ATiFL-DF was used instead of suture for augmented repair in this anatomical study. All specimens showed that the tibial origin of ATiFL-DF was successfully transferred to the talus insertion point of the upper bundle of ATFL, and the fibular origin of ATiFL-DF remained intact. The shortest distance between the anterolateral approach and the superficial peroneal nerve (SPN) was 3.8 mm, and the median distance was 3.9 mm. The anatomical experiment confirmed that the ATFL ATiFL-DF transfer augmentation repair under total arthroscopy was safe and feasible. The Department of Sports Medicine of our hospital has learned this operation early, and it has been applied in clinic. In our clinical work, we found that the above three surgical methods have good clinical efficacy, and the cost of operation is similar. However, we believe that ATiFL-DF transfer augmentation repair is more difficult and has a higher risk of complications because it changes the normal physiological anatomy. At present, as far as we know, there is a lack of relevant biomechanical research on ATFL suture augmentation repair, plus suture augmentation repair and ATiFL-DF transfer augmentation repair under total arthroscopy.

In our study, the failure tension and stiffness of the normal group were 148.2 ± 39.4 N and 12.1 ± 3.8 N/mm, respectively. Attarian et al. [17] performed biomechanical analysis on the lateral ligament of 12 ankle specimens in 1985 and reported for the first time that the failure tension of a normal ATFL was 138.9 ± 23.5 N. Waldrop III et al. [18] reported in 2012 that the failure tension of a normal ATFL was 160.9 ± 72.2 N and the stiffness was 12.4 ± 4.1 N/mm. Viens and Clanton et al. [19,20] reported in 2014 that the failure tension of a normal ATFL was 154.0 ± 63.7 N and the stiffness was 14.5 ± 4.4 N/mm. The failure tension and stiffness of our study are similar to those of normal ATFLs in other studies, which shows that the biomechanical research method used in our study is reliable.

### 4.1. Establishing ATFL Most Damage Model

Khawaji et al. [21] studied the anatomy of the ankles of 50 cadavers in 2015 and found that the average width of the ATFL was 4.97 mm. Chinese scholar Zhou Yunfeng et al. [22] analyzed the ankle specimens of 41 Chinese cadavers and found that the average width of a single bundle, double bundle and three bundles of ATFL were 6.91 ± 1.21 mm, 5.25 ± 0.79 mm and 3.94 ± 0.35 mm, respectively. This is the theoretical basis for our study to establish most injuries of ATFL, and the width of the ATFL we saw during operation is also between 5 and 6 mm. Through the scale at the end of the probe hook, we can successfully establish most ATFL injury models (residual ligament: 25–50%). In addition, we also performed MRI and B-ultrasound on ankle specimens before and after modeling, which verified the establishment of most ATFL injury models. In previous similar studies, most of the various surgical procedures were completed in ankle specimens with completely severed ATFL, while our study completed various surgical procedures in most ATFL injury models (residual ligament: 25–50%). In clinic, the disconnection of the ATFL often requires reconstruction surgery rather than repair surgery, although studies have shown that repair surgery can also achieve better clinical efficacy [23,24,25,26,27,28,29,30]. We believe that most ATFL injury models can better simulate the application of various surgical methods in clinic.

### 4.2. ATFL Plus Suture Augmentation Repair under Total Arthroscopy

The failure tension and stiffness of the plus suture augmentation group were 229.3 ± 66.7 N and 26.2 ± 8.2 N/mm, respectively, which were significantly higher than those of the normal group (148.2 ± 39.4 N, 12.1 ± 3.8 N/mm) and biological augmentation group (131.3 ± 38.8 N, 12.7 ± 5.2 N/mm), and the differences were statistically significant. At the same time, the average failure tension and stiffness of the plus suture augmentation group were also higher than those of the suture augmentation group (167.2 ± 47.2 N, 23.6 ± 7.0 N/mm), but the difference was not statistically significant. This study demonstrates that plus suture augmentation repair can enable patients to obtain higher tensile strength and rigidity than normal ATFL and ATiFL-DF transfer augmentation repair. This validates our clinical experience. In clinical practice, for some patients with high demand for exercise, we often choose this repair. At the same time, compared with many previous biomechanical studies of different surgical methods, the failure tension and stiffness of plus suture augmentation repair are also significantly higher. Waldrop III et al. [18] performed traditional Broström repair, fibular anchor augmentation Broström repair and talar anchor augmentation Broström repair in 18 ankle specimens, respectively. The failure tension and stiffness were (68.2 ± 27.8 N, 6.0 ± 2.5 N/m), (79.2 ± 34.3 N, 6.8 ± 2.7 N/mm) and (75.3 ± 45.6 N, 6.6 ± 4.0 N/mm), respectively, which were significantly lower than those in the plus suture enhanced repair in our study; it may be explained that the plus suture augmentation repair can obtain better tensile strength and rigidity than the traditional Broström repair, fibular anchor augmentation Broström repair and talar anchor augmentation Broström repair. Cottom et al. [20] performed single-row two-anchor Broström repair, double-row four-anchor Broström repair and double-row three-anchor Broström repair in 36 ankle specimens in 2016. The failure tension and stiffness were (156.43 ± 30.39 N, 12.10 ± 5.43 N/mm), (206.62 ± 55.62 N, 13.40 ± 7.98 N/mm) and (246.82 ± 82.37 N, 12.55 ± 4.00 N/mm), respectively. In this study, the failure tension of ATFL plus suture augmentation repair is higher than that of single-row two-anchor Broström repair, lower than that of double-row three-anchor Broström repair, which is similar to that of double-row four-anchor Broström repair. At the same time, the stiffness of ATFL plus suture augmentation repair was significantly higher than that of the above three methods. It may be stated that plus suture augmentation repair can obtain higher rigidity and tensile strength similar to Broström repair with single or double rows of anchors. Attarian et al. [17] performed allogeneic tendon reconstruction in six ankle specimens. The failure tension and stiffness were 170.7 ± 54.8 N and 23.1 ± 9.3 N/mm, respectively. In our study, the failure tension and stiffness of plus suture augmentation repair are higher than the former, which may indicate that plus suture augmentation repair may obtain better tensile strength and stiffness than allogeneic tendon reconstruction, but further research is still needed. Viens et al. [19] performed an open suture belt enhanced Broström operation and open suture belt enhanced operation to repair artificially broken ATFL in 12 ankle specimens. The failure tension and stiffness were (250.8 ± 122.7 N, 21.1 ± 9.1 N/mm) and (315.5 ± 66.8 N, 31.4 ± 9.9 N/mm), respectively. In our study, the failure tension and stiffness of plus suture augmentation repair are close to that of open suture belt enhanced repair, or it may be considered that plus suture augmentation repair can obtain tensile strength and stiffness close to that of suture belt enhanced repair. However, it should be noted that all the above previous studies were completed in ankle specimens with completely severed ATFLs, while our study was completed on most ATFL injury models (residual ligament: 25–50%). Therefore, the comparison with the previous research results still needs to be confirmed by further biomechanical research.

### 4.3. ATFL Suture Augmentation Repair under Total Arthroscopy

The failure tension and stiffness of suture augmentation repair were 167.2 ± 47.2 N and 23.6 ± 7.0 N/mm, respectively. Although the average value was higher than that of the normal group and biological augmentation group, it was not statistically significant. We cannot think that suture augmentation repair can obtain stronger tensile strength and rigidity than a normal ATFL, but we can think that suture augmentation repair can obtain tensile strength and rigidity similar to a normal ATFL, which can meet the needs of the vast majority of patients. Compared with other previous studies [17,18,19,20], suture augmentation repair can obtain significantly higher tensile strength and rigidity than traditional Broström repair, fibular anchor enhanced Broström repair and talus anchor enhanced Broström repair [18]. At the same time, suture augmentation repair can obtain tensile strength and higher rigidity similar to allogeneic tendon reconstruction [17], single-row or double-row anchor Broström repair [20]. In addition, suture augmentation repair can obtain rigidity similar to open suture belt augmentation repair [19]. It is also worth noting that all the above previous studies were completed in ankle specimens with completely severed ATFLs, while our study was completed on most ATFL injury models (residual ligament: 25–50%). Therefore, the results compared with previous studies still need to be confirmed by further biomechanical research.

### 4.4. ATFL ATiFL-DF Transfer Augmentation Repair under Total Arthroscopy

The failure tension and stiffness of biological augmentation repair were 131.3 ± 38.8 N and 12.7 ± 5.2 N/mm, respectively. The failure tension and stiffness were lower than those of plus suture augmentation repair and suture augmentation repair, and the difference was statistically significant. The failure tension and stiffness are similar to those of a normal ATFL, and the difference is not statistically significant. It can be seen that among the three surgical procedures, the failure tension and stiffness of the biological augmentation group are the lowest, which is close to a normal ATFL, but this can still meet the needs of most patients. Compared with other previous studies, ATiFL-DF transfer augmentation repair can obtain better tensile strength and rigidity than traditional Broström repair, fibular anchor enhanced Broström repair and talar anchor enhanced Broström repair [18]. As mentioned above, we believe that ATiFL-DF transfer augmentation repair is more difficult to perform and has a higher risk of complications because it alters the normal physiological anatomy. However, biomechanical experiments showed that this repair could not achieve better biomechanical strength, and we will be more careful in choosing this repair in the future. It should also be noted that the operations described in previous studies were performed in ankle specimens with completely severed ATFLs, whereas in our study, the operations were performed in most ATFL injury models (residual ligaments: 25–50%). Therefore, the results compared with previous studies still need to be confirmed by further biomechanical studies.

## 5. Conclusions

The tensile strength and rigidity of ATFL plus suture augmentation repair under total arthroscopy can be significantly better than that of normal ATFL and ATiFL-DF transfer augmentation repair. ATFL suture augmentation repair under total arthroscopy can obtain tensile strength similar to normal ATFL and ATiFL-DF transfer augmentation repair and can obtain rigidity significantly better than normal ATFL and ATiFL-DF transfer augmentation repair. ATFL ATiFL-DF transfer augmentation repair under total arthroscopy can achieve tensile strength and rigidity similar to a normal ATFL.

## Figures and Tables

**Table 1:** Comparison of failure tension and stiffness of three groups of ankle specimens.

Group	Failure Tension (N)	Stiffness (N/mm)
Normal group (*n* = 6)	148.2 ± 39.4	12.1 ± 3.8
Suture augmentation group (*n* = 6)	167.2 ± 47.2	23.6 ± 7.0
Plus suture augmentation group (*n* = 6)	229.3 ± 66.7	26.2 ± 8.2
Biological augmentation group (*n* = 6)	131.3 ± 38.8	12.7 ± 5.2

## Data Availability

The data from this study are available from the corresponding author upon request.
